# Upregulation of miR-361-3p suppresses serotonin-induced proliferation in human pulmonary artery smooth muscle cells by targeting SERT

**DOI:** 10.1186/s11658-020-00237-6

**Published:** 2020-10-07

**Authors:** Ying Zhang, Yongbin Chen, Guo Chen, Yingling Zhou, Hua Yao, Hong Tan

**Affiliations:** 1 Department of Cardiology, Guangdong Cardiovascular Institute, Guangdong Provincial People’s Hospital, Guangdong Academy of Medical Sciences, Guangdong 510080 Guangzhou, P. R. China; 2 Department of Cardiac Surgery, Guangdong Cardiovascular Institute, Guangdong Provincial People’s Hospital, Guangdong Academy of Medical Sciences, 510080 Guangzhou, P. R. China

**Keywords:** Abnormal proliferation, Pulmonary artery smooth muscle cells, Serotonin, miR-361-3p

## Abstract

**Background:**

Abnormal proliferation of pulmonary artery smooth muscle cells (PASMCs) is a key mechanism in pulmonary arterial hypertension (PAH). Serotonin (5-hydroxytryptamine, 5-HT) can induce abnormal proliferation of PASMCs. The role of miR-361-3p in serotonin-induced abnormal PASMCs proliferation remains unclear.

**Methods:**

The miR-361-3p level was analyzed in plasma from PAH patients and normal controls and in human PASMCs (hPASMCs) using RT-PCR. The hPASMCs were transfected with an miR-361-3p mimic and then treated with serotonin. Untransfected hPASMCs were used as the control. Cell proliferation was evaluated using an MTS assay and 5-ethynyl-2′-deoxyuridine (EdU) staining. The cell cycle stages were evaluated using flow cytometry. The association between miR-361-3p and serotonin transporter (SERT) was determined using a luciferase reporter assay and anti-AGO2 RNA immunoprecipitation assay. The protein expression was evaluated via western blotting.

**Results:**

The miR-361-3p level was lower in plasma from PAH patients than in plasma from the any of the normal control subjects. The mean pulmonary arterial pressure, pulmonary vascular resistance and pulmonary vascular resistance index were higher in PAH patients whose miR-361-3p level was lower than the median value for patients than in those whose miR-361-3p level was higher than the median. Serotonin treatment reduced miR-361-3p expression in the hPASMCs. MiR-361-3p overexpression suppressed cell proliferation, promoted apoptosis, induced G1 arrest, and decreased the phosphorylation level of ERK1/2 in serotonin-treated hPASMCs. SERT was identified as an miR-361-3p target. Its overexpression alleviated the effect of miR-361-3p overexpression on serotonin-induced hPASMC proliferation and upregulation of phosphorylated ERK1/2.

**Conclusions:**

The miR-361-3p level is lower in the plasma of PAH patients. Upregulation of miR-361-3p suppresses serotonin-induced proliferation of hPASMCs by targeting SERT. Our results suggest that miR-361-3p is a potential therapeutic target in PAH.

## Background

Pulmonary arterial hypertension (PAH) is associated with pathophysiological changes in the pulmonary arteries, including vasoconstriction, vascular remodeling due to abnormal proliferation of smooth muscle cells and abnormal aggregation of extracellular matrix, and in situ thrombosis. It results in a gradual increase in pulmonary vascular resistance that can cause right-sided heart failure and death [1]. PAH patients have a median survival time of only 2.8 years [2] and a five-year survival rate of only 50% [3]. Treatment outcomes remain unsatisfactory. Elucidating the mechanism of PAH progression would help identify novel diagnostic and therapeutic targets.

Pulmonary vascular remodeling is considered to be the main cause of PAH [4, 5]. Pulmonary artery smooth muscle cells (PASMCs) are the main cellular constituents of the pulmonary arterial walls and are located in the middle layer. A recent study suggests that abnormal proliferation of PASMCs is the key pathological basis for pulmonary vascular remodeling in PAH [6]. Their proliferation is promoted by vasoactive substances, such as serotonin (5-hydroxytryptamine; 5-HT) [7], which is a neurotransmitter involved in many physiological processes [8, 9]. Understanding the molecular mechanism of serotonin-induced abnormal PASMC proliferation and identifying effective therapeutic targets are key goals in PAH research.

MicroRNAs (miRNAs) play a crucial role in post-transcriptional regulation. For example, they inhibit mRNA translation into protein. They are also closely associated with various physiological processes in the cell [10]. Studies have reported on the potential of miRNAs as diagnostic biomarkers and therapeutic targets for PAH [11–14]. Several miRNAs are involved in pulmonary vascular remodeling [13]. Some promote the abnormal proliferation of PASMCs, whereas others suppress it [12, 14].

It is reported that miR-361-3p regulates proliferation, migration, invasion and stemness of cancer cells [15, 16]. In addition, it can suppress high glucose-induced inflammation and apoptosis of vascular endothelial cells [17]. It can also weaken cognitive deficits in Alzheimer's disease by inhibiting β-amyloid accumulation [18]. However, its role in the abnormal proliferation of PASMCs remains unclear.

This study aimed to establish its role in PAH. We evaluated the expression profile of miR-361-3p in the plasma of PAH patients and investigated the correlation with their clinical characteristics. We also analyzed the role and mechanism of miR-361-3p in serotonin-induced abnormal PASMC proliferation.

## Materials and methods

### Clinical samples

Forty patients with idiopathic PAH were consecutively enrolled from January 2017 to March 2018 at the Guangdong Provincial People's Hospital. Their clinical diagnosis of idiopathic PAH was according to the 2015 ESC/ERS guidelines for the diagnosis and treatment of pulmonary hypertension [1]. The exclusion criteria were:

1. Initiation of PAH-specific therapies before enrollment
2. Severe liver and kidney dysfunction
3. Other acute or chronic fatal diseases that could definitely worsen the prognosis
4. Inability to sign informed consent or cooperate with the researchers

Twenty normal volunteers matched for age, gender and race were enrolled simultaneously. Written informed consent was signed by all participants. The study was approved by the Ethics Committee of the Guangdong Provincial People’s Hospital (No. GDREC2016305H).

Right-side heart catheterization was performed following admission. The values of the mean pulmonary arterial pressure, pulmonary vascular resistance, and pulmonary vascular resistance index of the PAH patients were recorded. Plasma was collected and stored at – 80 °C for subsequent assays.

### RNA extraction and quantitative RT-PCR

A HiPure Liquid RNA/miRNA Kit (R4163-02; MAGEN, China) was used to extract total RNA from the plasma. The RNA (1 µg) was reverse transcribed using MMLV Reverse Transcriptase (RT; Promega, USA). The RT primer for miR-361-3p was 5′-CTCAACTGGTGTCGTGGAGTCGGCAATTCAGTTGAGAAATCAGA-3′ and for the internal reference gene U6 was 5′-AACGCTTCACGAATTTGCGT-3′. The qPCR mix was prepared using SYBR Green qPCR SuperMix (Invitrogen, USA) and qPCR was performed on an ABI PRISM 7500 Real-Time PCR System (Applied Biosystems; Thermo Fisher Scientific, USA). The forward primer for miR-361-3p was 5′-ACACTCCAGCTGGGTCCCCCAGGTGTGATTCTG-3′ and the reverse primer was 5′-CTCAACTGGTGTCGTGGA-3′. The forward primer for U6 was 5′-CTCGCTTCGGCAGCACA-3′ and the reverse primer was 5′-AACGCTTCACGAATTTGCGT-3′. The relative expression of miR-361-3p was calculated using the 2^−ΔΔCt^ method [19].

### Cell culture

Human PASMCs (hPASMCs) were purchased from ScienCell Research Laboratories (USA) and cultured in smooth muscle cell medium (cat. no. 1101, ScienCell Research Laboratories) at 37 °C in a humidified incubator infused with air (21% O_2_ and 5% CO_2_).

### Preparation of the miR-361-3p mimic and construction of the serotonin transporter (SERT) overexpression plasmid

Negative control miRNA (miR-NC), miR-361-3p mimic (miR-361-3p), miR-NC inhibitor and miR-361-3p inhibitor were purchased from GenePharma (China). To construct the SERT overexpression plasmid (ov-SERT), the full coding sequence of SERT was cloned into plasmid pcDNA3.1 + at the KpnI and XhoI sites, using the following primers: 5′-cggggtaccgccaccATGGAGACGACGCCCTTGAATTCTCAG-3′ and 5′-ccgctcgagTTACACAGCATTCAAGCGGATGTCCCCACA-3′. Empty plasmid pcDNA3.1 + was used as a negative control (pCDNA).

### Serotonin treatment, transient transfection and cell groups

To investigate the effect of serotonin on the proliferation of hPASMCs, cells were treated with 0, 50, 100, 250, 500 and 1000 μmol/l serotonin for 0, 24, 48 and 72 h. To investigate the effect of miR-361-3p on serotonin treated-hPASMCs, cells were divided into four groups: blank (no treatment), serotonin (treated with 250 μmol/l serotonin for 48 h), serotonin + miR-NC (transfected with miR-NC for 24 h, then treated with 250 μmol/l serotonin for 48 h), serotonin + miR-361-3p group (transfected with miR-361-3p mimic for 24 h, then treated with 250 μmol/l serotonin for 48 h).

To investigate whether SERT overexpression weakens the effect of miR-361-3p overexpression, the serotonin-treated-hPASMCs were divided into three groups: miR-NC + pcDNA (transfected with miR-NC and empty pcDNA3.1 + plasmid), miR-361-3p + pcDNA (transfected with miR-361-3p mimic and empty pcDNA3.1 + plasmid), and miR-361-3p + ov-SERT group (transfected with miR-361-3p mimic and ov-SERT). The transient transfections were performed using Lipofectamine 2000 (Invitrogen).

### MTS assay

The CellTiter 9 AQ_ueous_ One Solution Cell Proliferation Assay (MTS, Promega) was used to assess the effect of the various treatments on cell proliferation. Briefly, CellTiter 96 AQ_ueous_ One Solution reagent (20 μl) was added to the culture medium at the end of the experimental period and incubated in a humidified incubator (21% O_2_ and 5% CO_2_) for 2 h. The absorbance was measured at an optical density (OD) of 490 nm using a Multiskan MK3 microplate reader (Thermo Fisher Scientific). The rate of cell proliferation was calculated as: proliferation rate = (experimental OD value – blank OD value)/(control OD value – blank OD value) × 100%.

### 5-ethynyl-2′-deoxyuridine (EdU) staining

Following the treatments described in the methods section, the cells were incubated in culture medium containing 50 μM EdU (Solarbio, China) for 2 h. Next, the cells were fixed by successive incubation with 4% paraformaldehyde (Merck KGaA, Germany) in phosphate-buffered saline (PBS) for 20 min, 2 mg/ml glycine (Merck KGaA) for 10 min, and 0.5% TritonX-100 (Merck KGaA) in PBS for 10 min. After washing with PBS once, 1× Apollo staining solution (Solarbio) was added to each well to detect the EdU signal and incubated for 30 min. After decolorization with 0.5% TritonX-100 in PBS, the cells were washed twice with methyl alcohol and once with PBS, and then stained with 1× Hoechst 33342 solution (Solarbio) for 30 min to stain the nuclei. After washing with PBS three times, five randomly selected fields were photographed with a fluorescence microscope (EVOS FL Auto Cell Imaging System; Thermo Fisher Scientific). The numbers of EdU-positive and Hoechst 33342-stained cells were counted using Image Pro-Plus 6.0 (Media Cybernetics, USA). The percentage of EdU-positive cells was calculated as: (number of EdU-positive cells/number of Hoechst 33342-stained cells) × 100%.

### Cell cycle assay

Harvested cells were suspended in 300 μl PBS, then 700 μl absolute ethanol was slowly added to fix the cells. After fixing at – 20 °C overnight, the cell suspension was centrifuged at 2000 rpm for 10 min, and the supernatant was discarded. After washing with PBS, the cell pellet was stained with 300 μl propidium iodide solution at 37 °C in the dark for 15 min.

### Western blotting

Total cellular protein extraction, protein concentration quantification and western blotting were performed as described in our previous report [20]. Briefly, proteins were transferred onto a polyvinylidene difluoride (PVDF) membrane after separation using sodium dodecyl sulfate polyacrylamide gel electrophoresis (SDS-PAGE). After blocking with 5% non-fat milk (5 g/100 ml PBS containing 0.1% Tween-20) at 25 °C for 2 h, the PVDF membranes were separately incubated with primary antibodies at 4 °C overnight. The next day, the PVDF membranes were washed to remove unbound primary antibodies and then incubated with the secondary antibody. Finally, after washing to remove unbound secondary antibodies, the chemiluminescent signal was detected via exposure to X-rays. The primary antibodies used in western blotting were: anti-cyclin D1 (1:1000, ab226977), anti-cyclin E1 (1:2,000, ab71535), anti-SERT (1:800, ab102048), anti-total extracellular-regulated kinase (ERK)1/2 (t-ERK1/2, 1:1000, ab17942), anti-phosphorylated ERK1/2 (p-ERK1/2, 1:800, ab2143620), anti-cleaved caspase-9 (1:1000, ab2324), anti-Bcl-2 (1:2000, ab196495), and anti-GAPDH (1:3000, ab9485). All the primary antibodies were purchased from Abcam (USA). GAPDH was used as a loading control.

### Luciferase reporter assay

miR-361-3p-binding sites in the 3′-untranslated region (UTR) of SERT messenger RNA were predicted using TargetScan (human 7.2 version, https://www.targetscan.org/). To construct the luciferase reporter, wild-type SERT 3′-UTR was cloned into the psi-CHECK-2 vector (Promega), using the following primers: 5′-ccgctcgagCACACTCACCGAGAGGAAAAAGGCTTCTCC-3′ (forward) and 5′-ataagaatgcggccgcTTCACAGCATAAATCATTTATTAATATC-3′ (reverse). PCR-based site-directed mutagenesis was performed to mutate the miR-361-3p-binding sites in wild-type luciferase reporter using the following primers: 5′-GAATTTTGTCGTTGAAAAACGAGAATAGATGGCATCAGTCCTTCAATTCTGTAACT-3′ (forward) and 5′-CATCTATTCTCGTTTTTCAACGACAAAATTCTTCTTAGTTCAGTAGACATTCAAAC-3′ (reverse). The wild-type and mutant luciferase reporters were called wild-type 3′-UTR and mutant 3′-UTR, respectively.

Human embryonic kidney 293 T cells were plated in 24-well plates and co-transfected with 0.5 µg wild-type 3′-UTR plasmid along with 50 nM miR-361-3p mimic, 0.5 μg wild-type 3′-UTR plasmid with 50 nM miR-NC, 0.5 μg mutant 3′-UTR plasmid with 50 nM miR-361-3p mimic, or mutant 3′-UTR plasmid with 50 nM miR-NC using Lipofectamine 2000. The activity of firefly luciferase or *Renilla* luciferase was measured 48 h post-transfection using a Dual-Luciferase Assay kit (Promega). The relative luciferase activity was expressed as a ratio of *Renilla* luciferase to firefly luciferase.

### Anti-AGO2 RNA immunoprecipitation (RIP) assay

hPASMCs were transfected with miR-361-3p mimic or miR-NC. After transfection for 48 h, cells were harvested and resuspended in 100 µl cell lysis buffer for immunoprecipitation (Beyotime Biotechnology, China) containing protease and RNase inhibitors. The RIP assay was performed following the instructions for the Magna RIP RNA-Binding Protein Immunoprecipitation Kit (Millipore, USA). The antibodies anti-AGO2 and anti-IgG were purchased from Abcam. Finally, the SERT mRNA level in the RIP products was analyzed using quantitative RT-PCR. The cell lysate before immunoprecipitation was termed the ‘input’ group. The products of the RIP assay targeting AGO2 or IgG were named the AGO2 group or IgG group, respectively.

### Statistical analysis

Statistical analysis was performed using GraphPad Prism version 7.0 (GraphPad software, USA). The expression levels of miR-361-3p in the plasma from PAH patients and normal controls were described using the median and the 25th and 75th percentiles. All the data pertaining to miR-361-3p expression profiles in the plasma were analyzed using the Mann–Whitney U test. Results from three independent experiments are expressed as means ± standard deviation. Statistical differences for the cell experiments were evaluated using one-way analysis of variance. p < 0.05 was considered statistically significant.

## Results

### miR-361-3p expression profile in the plasma of PAH patients

**Fig. 1:**
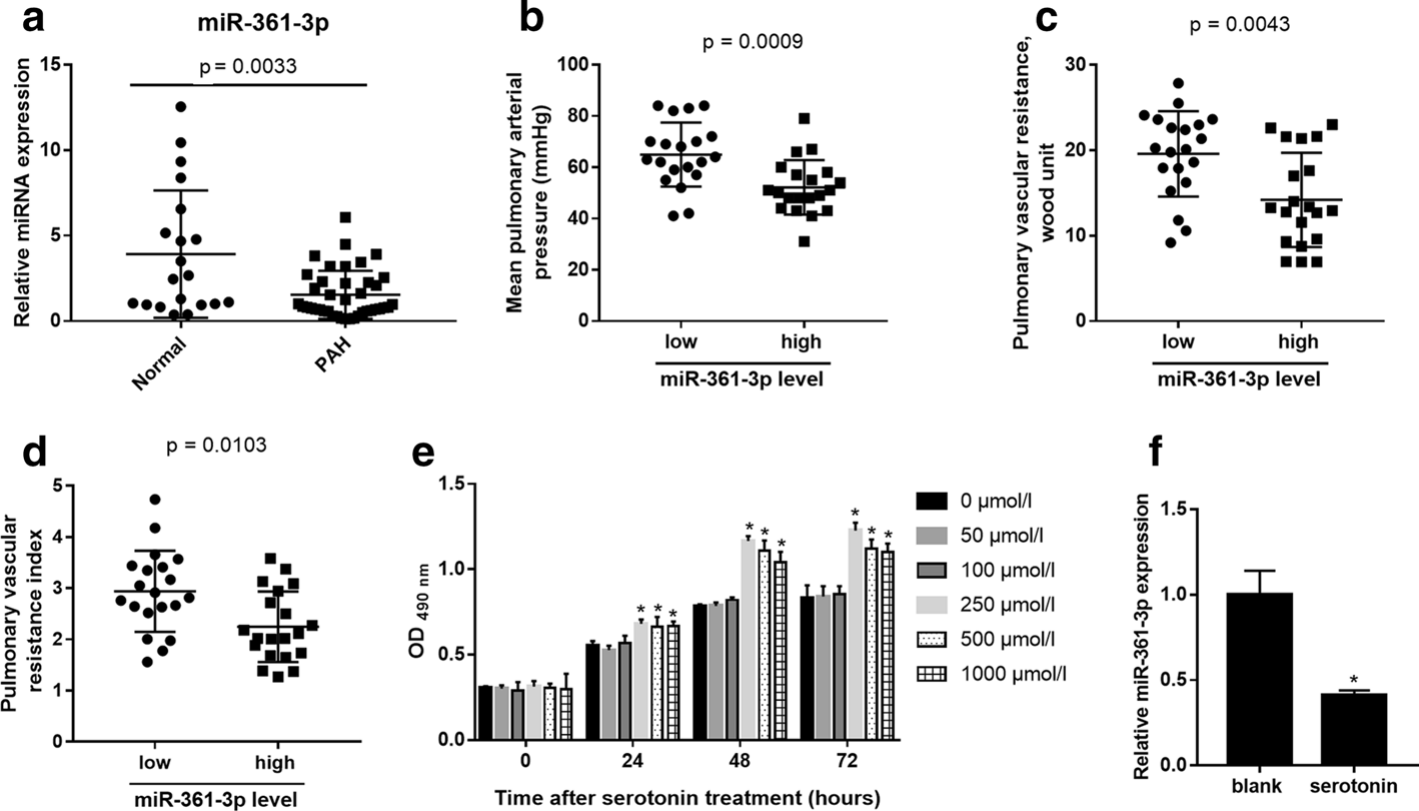
The miR-361-3p expression profile in the plasma of PAH patients and in hPASMCs treated with serotonin. **a** Relative expression level of miR-361-3p in the plasma of PAH patients and normal control subjects. Correlation of miR-361-3p with mean pulmonary arterial pressure (**b**), pulmonary vascular resistance (**c**) and pulmonary vascular resistance index (**d**). **e** The OD_490nm_ value of PASMCs treated with 0, 50, 100, 250, 500 and 1000 μmol/l serotonin for 24, 48 and 72 h. The MTS assay was performed and the OD_490nm_ was measured. *p < 0.05, when compared to results for 0 μmol/l. **f** miR-361-3p level in hPASMCs treated with 250 μmol/l serotonin (serotonin) or not treated (blank) for 48 h. The miR-361-3p level was determined using quantitative RT-PCR. *p < 0.05, serotonin group vs. blank group

The relative expression level of miR-361-3p in the plasma was higher for the PAH patients than for the normal controls (median [25th and 75th percentiles]: PAH: 0.83 [0.585, 6.07]; controls: 2.565 [0.97, 12.54]; p = 0.0033; Fig. 1a). To analyze the correlation of the miR-361-3p levels with clinical parameters, PAH patients were divided into two groups based on the median miR-361-3p level and are hereafter referred to as the high level (above the median) and low level (below the median) groups.

The mean values of pulmonary arterial pressure, pulmonary vascular resistance and pulmonary vascular resistance index were higher for PAH patients with low miR-361-3p levels than for those with high levels (Fig. 1b through d). This expression profile suggests that miR-361-3p may be involved in the development and progression of PAH.

### miR-361-3p level is reduced by serotonin in hPASMCs

hPASMCs were treated with different concentrations of serotonin for 24, 48 and 72 h. After an MTS assay, the OD_490nm_ of each hPASMC group was measured. The OD_490nm_ value of hPASMCs treated with 250, 500 and 1000 μmol/l serotonin for 24, 48 and 72 h was significantly higher than that of hPASMCs not treated with serotonin (Fig. 1e), indicating that serotonin treatment increases the proliferation of hPASMCs. We found that the 250 μmol/l serotonin treatment had the most significant effect on the abnormal proliferation of PASMCs, so this treatment was used to induce abnormal proliferation in subsequent assays.

hPASMCs were treated with 250 μmol/l serotonin for 48 h and harvested for quantitative RT-PCR analysis of miR-361-3p levels. The miR-361-3p level was significantly lower in the serotonin group than in the blank group (Fig. 1f). These results indicate that serotonin treatment reduces miR-361-3p levels in hPASMCs.

### miR-361-3p overexpression suppresses serotonin-induced hPASMC proliferation

To further examine the effect of miR-361-3p on serotonin-treated-hPASMCs, the miR-361-3p levels were analyzed in hPASMCs in the blank, serotonin, serotonin + miR-NC, and serotonin + miR-361-3p groups. Cells were harvested for quantitative RT-PCR following 48 h treatment with serotonin. Our results indicate that the miR-361-3p level was significantly higher in hPASMCs in the serotonin + miR-361-3p group than in those in the serotonin + miR-NC group, indicating successful overexpression of miR-361-3p in that group of cells (Additional file 1).

**Fig. 2:**
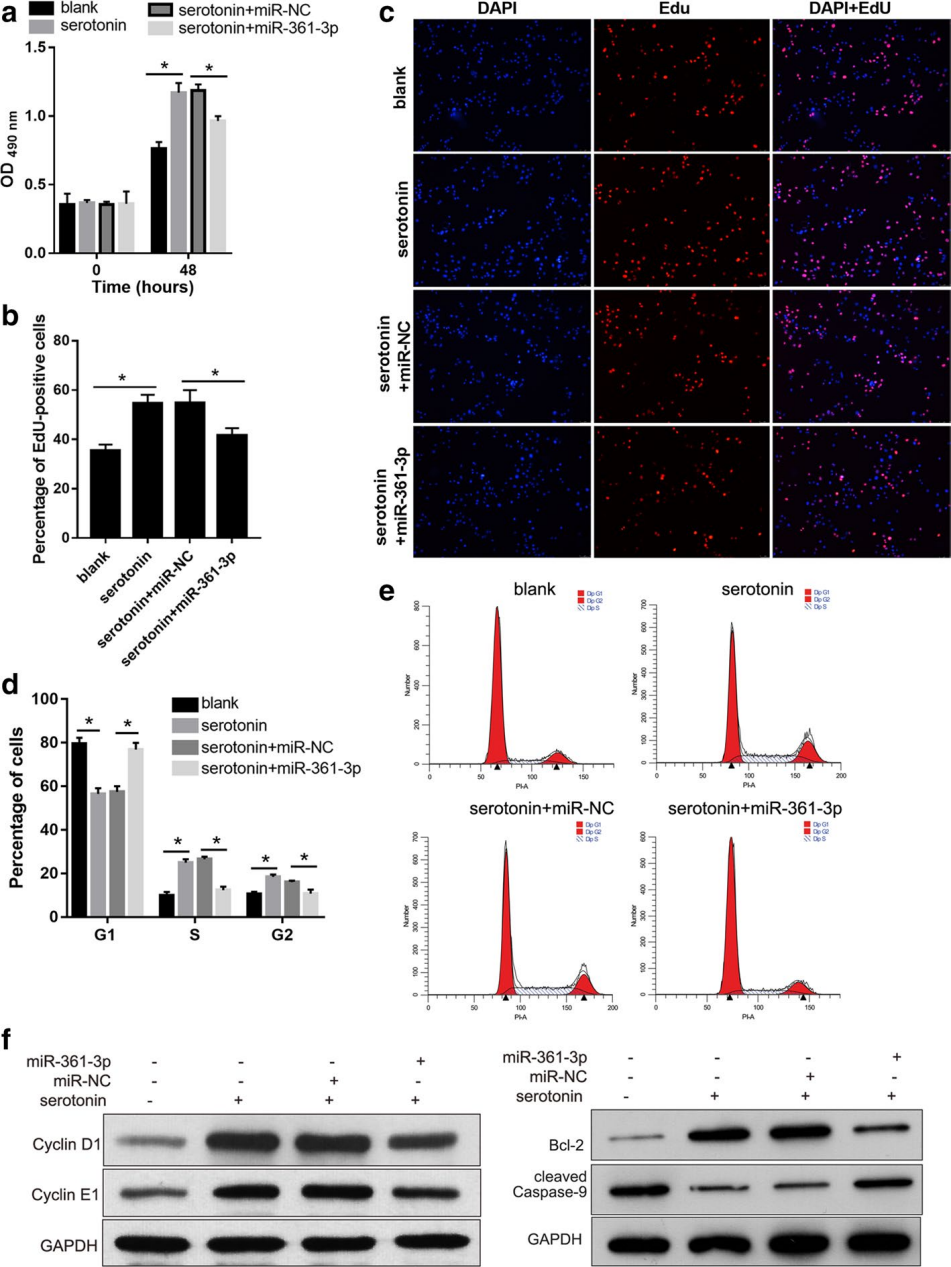
miR-361-3p overexpression suppresses serotonin-induced cell proliferation of hPASMCs. hPASMCs were divided into four groups: blank (no treatment), serotonin (treated with 250 μmol/l serotonin for 48 h), serotonin + miR-NC (after transfection with miR-NC for 24 h, treated with 250 μmol/l serotonin for 48 h), and serotonin + miR-361-3p group (after transfection with miR-361-3p mimic for 24 h, treated with 250 μmol/l serotonin for 48 h). **a** Results of MTS assay showing the OD_490nm_ of the four groups. **b** Results of EdU staining showing the percentage of EdU-positive cells. **c** Representative images of EdU staining from the four groups. **d** Graphical representation of the percentage of cells in the G1-, S- and G2-phases. **e** Representative images of the cell cycle assay. **f** Cyclin D1, cyclin E1, cleaved caspase-9 and Bcl-2 levels were determined using western blot analysis. *p < 0.05

Next, we investigated the effect of miR-361-3p overexpression on serotonin-induced hPASMC proliferation using the MTS assay and EdU staining (Fig. 2a through c). We found that the OD_490nm_ value and percentage of EdU-positive cells in the serotonin group were higher than those in the blank group. There was no obvious difference between the serotonin group and serotonin + miR-NC group. The OD_490nm_ value and percentage of EdU-positive cells in the serotonin + miR-361-3p group was lower than that in the serotonin + miR-NC group. These results indicate that miR-361-3p overexpression suppresses serotonin-induced hPASMC proliferation.

To determine the mechanism by which miR-361-3p overexpression suppresses serotonin-induced hPASMC proliferation, we analyzed the distribution of the cells in various stages of the cell cycle using flow cytometry and determined the expression of cell cycle-related proteins (cyclin D1 and cyclin E1) and apoptosis-related proteins (cleaved caspase-9 and Bcl-2) in the four groups of cells. The percentage of cells in the G1-phase was lower in the serotonin group than that in the blank group, whereas the percentage of cells in the S- and G2-phases was higher in the serotonin group than that in the blank group (Fig. 2d, e). There were no obvious differences between the serotonin and serotonin + miR-NC groups. The percentage of cells in the G1-phase was higher in the serotonin + miR-361-3p group than that in the serotonin + miR-NC group, whereas the percentage of cells in S- and G2-phase was lower in the serotonin + miR-361-3p group than that in serotonin + miR-NC group (Fig. 2d, e). Moreover, we found that Bcl-2, cyclin D1 and cyclin E1 expression levels were higher in the serotonin group than those in the blank group, whereas their expression levels were lower in the serotonin + miR-361-3p group than those in the serotonin + miR-NC group (Fig. 2f). The level of cleaved caspase-9 was lower in the serotonin group than that in the blank group, whereas it was higher in the serotonin + miR-361-3p group than that in the serotonin + miR-NC group (Fig. 2f). These results indicate that miR-361-3p overexpression induces G1-phase arrest and promotes apoptosis in serotonin-treated PASMCs.

### SERT is a target of miR-361-3p

**Fig. 3:**
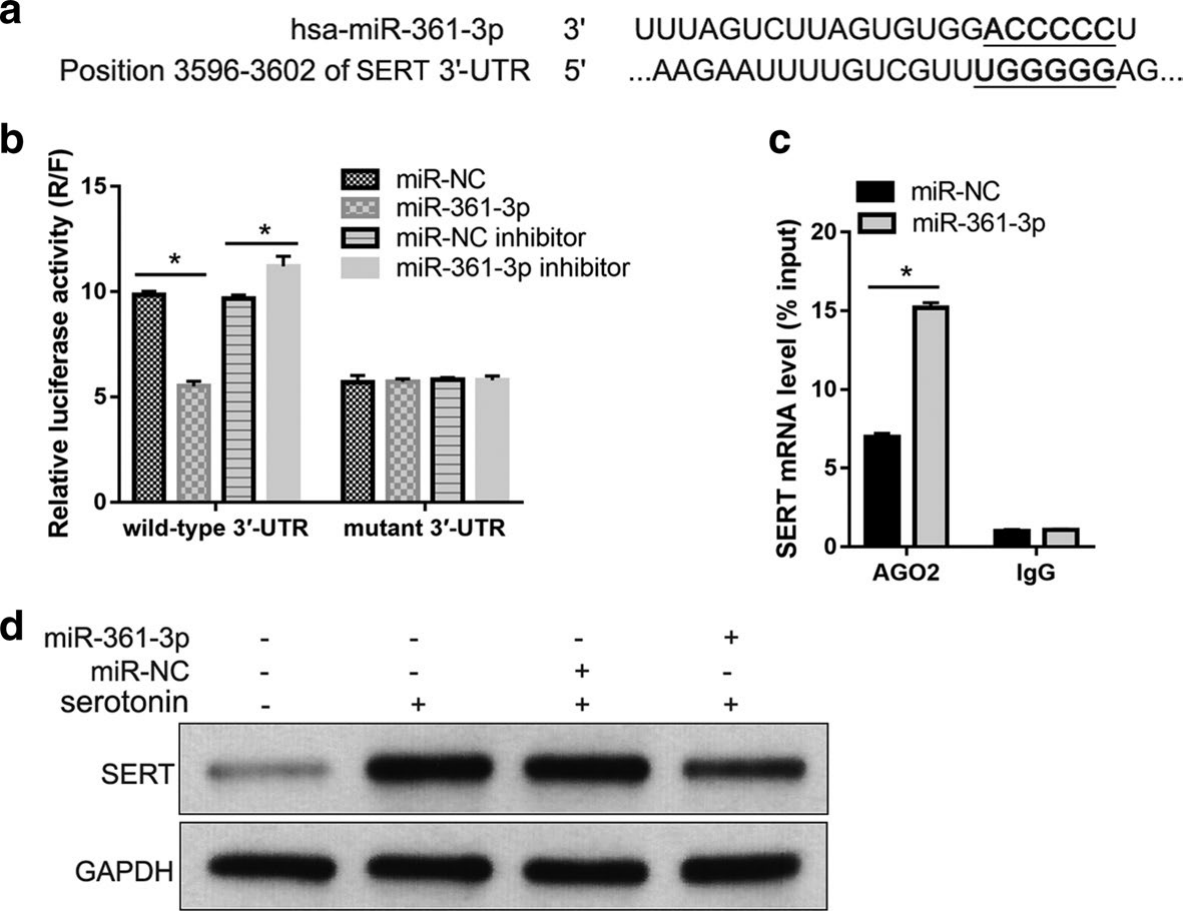
SERT is a target of miR-361-3p. **a** The binding site of miR-361-3p in the 3′-UTR of SERT. **b** Results of luciferase reporter assay. 293 T cells were co-transfected with wild type 3′-UTR plasmid and miR-361-3p mimic, wild type 3′-UTR plasmid with miR-NC, mutant 3′-UTR plasmid with miR-361-3p mimic, or mutant 3′-UTR plasmid with miR-NC. After transfection for 48 h, the activities of firefly and *Renilla* luciferase were measured and relative luciferase activity is expressed as the ratio of *Renilla* luciferase to firefly luciferase (R/F). **c** SERT mRNA level in RIP products analyzed using quantitative RT-PCR. The cell lysate before immunoprecipitation was termed the ‘input’ group. The products of a RIP assay targeting AGO2 or IgG were named the AGO2 group or IgG group. **d** SERT level in blank (no treatment), serotonin (treated with 250 μmol/l serotonin for 48 h), serotonin + miR-NC (after transfection with miR-NC for 24 h, treated with 250 μmol/l serotonin for 48 h), and serotonin + miR-361-3p (after transfection with miR-361-3p mimic for 24 h, treated with 250 μmol/l serotonin for 48 h) groups. *p < 0.05

We used the TargetScan database to predict the targets of miR-361-3p. SERT stood out among the predicted targets. Also called solute carrier family 6 member 4, SERT is a transmembrane transporter with a high affinity for serotonin [21]. The binding site of miR-361-3p in the 3′-UTR of SERT is shown in Fig. 3a.

The results of the luciferase reporter assay show that co-transfection with the miR-361-3p mimic decreased the relative luciferase activity of wild type 3′-UTR compared to its activity after co-transfection with miR-NC. Co-transfection with the miR-361-3p inhibitor increased the relative luciferase activity of wild type 3′-UTR compared to its activity after co-transfection with the miR-NC inhibitor (Fig. 3b). The results of the anti-AGO2 RIP assay show that the SERT mRNA level was higher in AGO2 RIP products of PASMCs transfected with the miR-361-3p mimic than in PASMCs transfected with miR-NC (Fig. 3c).

These results indicate that miR-361-3p binds to the 3′-UTR of SERT mRNA. Moreover, we found that the expression level of SERT was higher in the serotonin group than that in the blank group, whereas SERT expression was lower in the serotonin + miR-361-3p group than that in the serotonin + miR-NC group (Fig. 3d).

### SERT overexpression alleviated the effect of miR-361-3p overexpression on serotonin-induced cell proliferation of hPASMCs

**Fig. 4:**
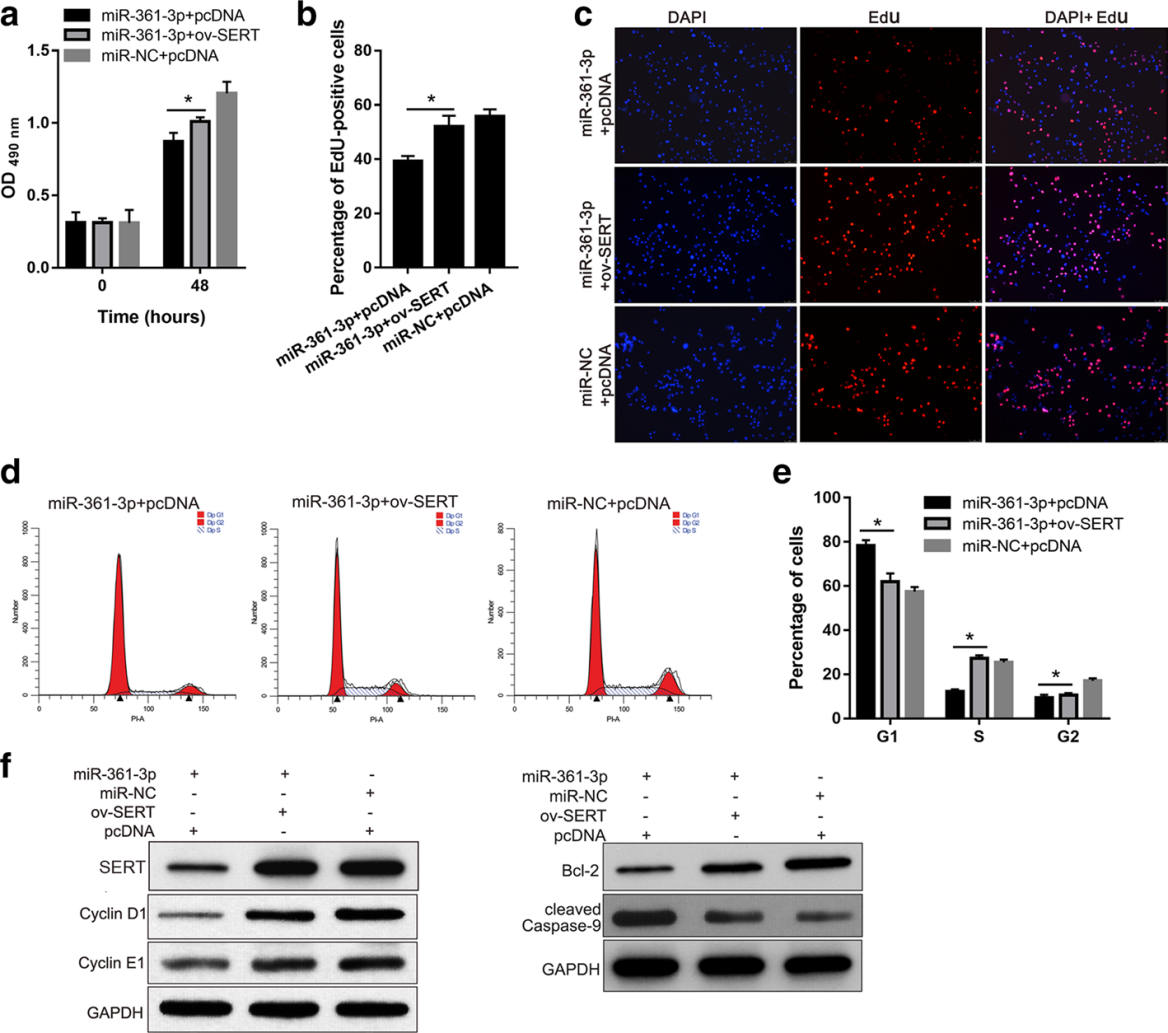
SERT overexpression alleviates the effect of miR-361-3p overexpression on serotonin-induced cell proliferation of PASMCs. serotonin treated-hPASMCs were divided into three groups: miR-NC + pcDNA (transfected with miR-NC and empty pcDNA3.1 + plasmid), miR-361-3p + pcDNA (transfected with miR-361-3p mimic and empty pcDNA3.1 + plasmid), and miR-361-3p + ov-SERT (transfected with miR-361-3p mimic and ov-SERT). **a** Results of the MTS assay showing the OD_490nm_ for the three groups. Results of EdU staining with the percentage of EdU-positive cells (**b**) and representative images of EdU staining from the three groups (**c**). **d** Results of the cell cycle assay with representative images from the three groups (**d**) and a graphical representation of the percentage of cells at the G1-, S- and G2-phases (**e**). **f** Level of SERT, cyclin D1 and cyclin E1 expressions determined using western blot analysis. GAPDH served as the loading control. *p < 0.05

The next experiments were designed to further confirm that miR-361-3p overexpression suppresses serotonin-induced cell proliferation of hPASMCs by decreasing the SERT protein level. SERT was overexpressed to reverse the effect of miR-361-3p in serotonin-treated hPASMCs following co-transfection with the miR-361-3p mimic and ov-SERT. We examined cell proliferation, cell cycle and apoptosis in hPASMCs of the miR-NC + pcDNA, miR-361-3p + pcDNA, and miR-361-3p + ov-SERT groups. The results show that the OD_490nm_ value and percentage of EdU-positive cells were higher in the miR-361-3p + ov-SERT group than those in the miR-361-3p + pcDNA group (Fig. 4a through c). The percentage of cells in the G1-phase in the miR-361-3p + ov-SERT group was lower than that in the miR-361-3p + pcDNA group, whereas the percentage of cells in the S- and G2-phases in the miR-361-3p + ov-SERT group was higher than that in the miR-361-3p + pcDNA group (Fig. 4d, e). Moreover, we found that the expression levels of cyclin D1, cyclin E1 and Bcl-2 were higher, whereas that of cleaved caspase-9 level was lower in the miR-361-3p + ov-SERT group than that in the miR-361-3p + pcDNA group (Fig. 4f). In addition, the expression level of SERT in the miR-361-3p + ov-SERT group was higher than that in the miR-361-3p + pcDNA group, but it was almost the same as that of the pcDNA + miR-NC group, indicating that ov-SERT transfection successfully reversed the effect of miR-361-3p transfection on SERT expression (Fig. 4f). These results indicate that SERT overexpression alleviates the effect of miR-361-3p overexpression on serotonin-induced hPASMC proliferation.

### The effect of the miR-361-3p/SERT axis on serotonin-induced upregulation of p-ERK1/2

**Fig. 5:**
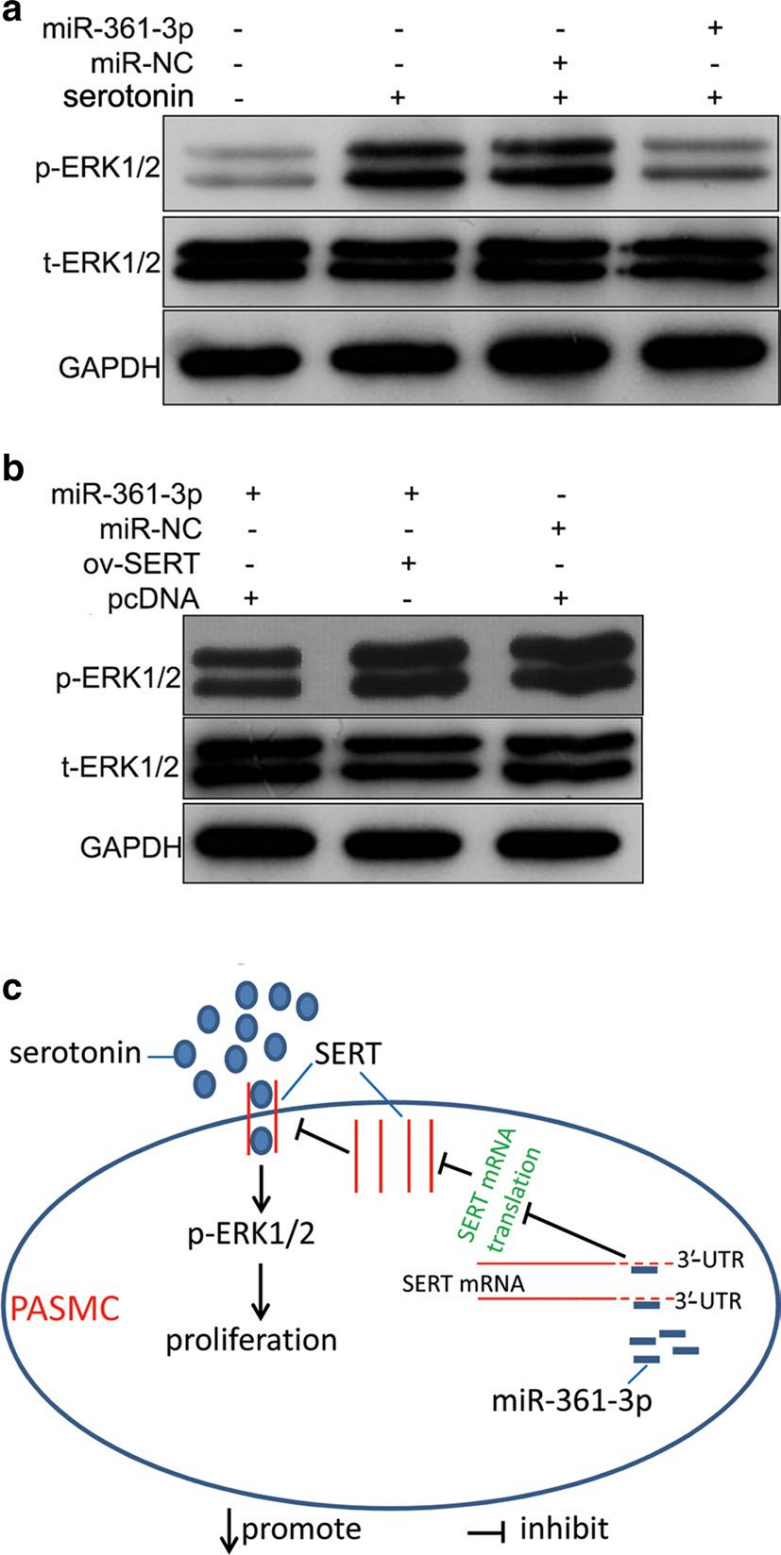
Effect of the miR-361-3p/SERT axis on serotonin-induced upregulation of p-ERK1/2 and the signaling diagram of this study. **a** The level of t-ERK1/2 and p-ERK1/2 in the hPASMCs of the blank group (no treatment), serotonin group (treated with 250 μmol/l serotonin for 48 h), serotonin + miR-NC group (after transfection with miR-NC for 24 h, treated with 250 μmol/l serotonin for 48 h), and serotonin + miR-361-3p group (after transfection with miR-361-3p mimic for 24 h, treated with 250 μmol/l serotonin for 48 h). **b** The level of t-ERK1/2 and p-ERK1/2 in the hPASMCs of the miR-NC + pcDNA group (transfected with miR-NC and empty pcDNA3.1 + plasmid), miR-361-3p + pcDNA group (transfected with miR-361-3p mimic and empty pcDNA3.1 + plasmid), and miR-361-3p + ov-SERT group (transfected with miR-361-3p mimic and ov-SERT). **c** The signaling diagram indicates that miR-361-3p plays its role by suppressing the translation of SERT mRNA to reduce the intracellular accumulation of serotonin, consequently inhibiting the ERK1/2 signal

To explore the mechanism underlying the effect of the miR-361-3p/SERT axis on serotonin-induced hPASMC proliferation, we determined the levels of t-ERK1/2 and p-ERK1/2. The p-ERK1/2 level was higher in the serotonin group than that in the blank group, but lower in the serotonin + miR-361-3p group than that in the serotonin + miR-NC group (Fig. 5a). Moreover, the level of p-ERK1/2 in the miR-361-3p + ov-SERT group was higher than that in the miR-361-3p + pcDNA group, but it was almost the same as that in the pcDNA + miR-NC group (Fig. 5b). The level of t-ERK1/2 showed no obvious change between these groups (Fig. 5a, b).

## Discussion

We investigated the potential role of miR-361-3p in PAH. First, we examined the expression profile of miR-361-3p in the plasma of PAH patients and analyzed its correlation with clinical parameters. We found that the miR-361-3p level was higher in the plasma of PAH patients than in that of the normal control subjects. Moreover, the values of the mean pulmonary arterial pressure, pulmonary vascular resistance and pulmonary vascular resistance index were higher in PAH patients whose miR-361-3p level was lower than the median value for patients than in those whose miR-361-3p level was higher than the median. These results indicate that miR-361-3p may play a role in the development and progression of PAH. Next, we designed experiments to further investigate the role of miR-361-3p in PAH.

As discussed in the introduction, abnormal proliferation of PASMCs is one of the main mechanisms involved in pulmonary vascular remodeling in PAH [6]. Increased synthesis and/or activity of serotonin in pulmonary arteries is known to be involved in the pathobiology of PAH and serotonin can cause abnormal proliferation of PASMCs [22, 23]. Therefore, we constructed an in vitro hPASMC model with abnormal proliferation induced by serotonin to determine the therapeutic potential of miR-361-3p in PAH.

We found that the miR-361-3p level was downregulated in the serotonin-treated hPASMCs. This suggests that miR-361-3p expression or degradation is controlled by serotonin and is consistent with the low miR-361-3p expression observed in the plasma of PAH patients. We hypothesized that miR-361-3p overexpression might suppress serotonin-induced hPASMC proliferation. Successful miR-361-3p overexpression was achieved in hPASMCs via transfection with a mimic. The results of MTS assays and EdU staining revealed that miR-361-3p overexpression suppressed proliferation, induced G1-phase arrest, decreased the level of the anti-apoptotic protein Bcl-2, and increased the level of the pro-apoptotic protein cleaved caspase-9 in the serotonin-treated hPASMCs. These findings indicate that miR-361-3p could inhibit the cell cycle and promote apoptosis in serotonin-treated hPASMCs. Taken together, these results suggest that miR-361-3p is a potential therapeutic target to suppress the abnormal proliferation of hPASMCs and consequently improve pulmonary vascular remodeling in PAH.

Among the potential miR-361-3p target genes that were predicted using TargetScan, we focused on SERT, which has a high affinity for serotonin [21]. SERT transports serotonin into PASMCs to promote cell division and causes their abnormal proliferation [23, 24]. The SERT inhibitor fluoxetine can decrease the proliferation rate of PASMCs treated with serotonin [25], suggesting that the inhibition of serotonin uptake by its transporter may be a means to suppress the proliferation of PASMCs.

We hypothesized that miR-361-3p suppresses serotonin-induced cell proliferation of hPASMCs by inhibiting SERT mRNA translation. The results of our luciferase reporter assay and anti-AGO2 RIP assay showed that miR-361-3p binds to the 3′-UTR of SERT. In addition, we found that miR-361-3p overexpression decreases the upregulation of SERT induced by serotonin treatment in hPASMCs. Thus, SERT is a target of miR-361-3p. Furthermore, we found that SERT overexpression alleviates the effect of miR-361-3p overexpression on serotonin-induced cell proliferation of hPASMCs. All these results reveal that miR-361-3p overexpression suppresses serotonin-induced hPASMC proliferation by decreasing the SERT protein level (Fig. 5c).

Serotonin affects the proliferation of PASMCs through serotonin receptors or SERT [9]. The underlying mechanism of serotonin receptors or SERT is not the same. Both are integral membrane proteins. Extracellular serotonin plays its role by activating serotonin receptors, such as 5-HT1B [18]. In addition, it can be transported into PASMCs by SERT. Intracellular accumulation of serotonin or activation of the serotonin receptors leads to the phosphorylation and nuclear translocation of ERK1/2 [24]. Therefore, we evaluated the effect of miR-361-3p on the ERK1/2 signal. We found that miR-361-3p overexpression can decrease the phosphorylation of ERK1/2, and this decrease can be alleviated by SERT overexpression. These results further suggest that miR-361-3p may play its role by suppressing SERT to reduce the intracellular accumulation of serotonin, consequently inhibiting the ERK1/2 signal (Fig. 5c). In future studies, we will focus on the effect of miR-361-3p on serotonin receptors and the underlying mechanism of this interaction.

Our study also has some limitations. First, although the miR-361-3p level in the plasma of PAH patients was elevated, more clinical cases are needed to analyze whether miR-361-3p can be used as a diagnostic marker of PAH. Second, we did not verify the role of miR-361-3p in vivo using animal models. Third, it is clear that SERT is not the only target gene of miR-361-3p, and it is possible that miR-361-3p may mediate its effect through other target genes.

## Conclusions

Our findings show that the miR-361-3p level was lower in the plasma of PAH patients than in that of normal control subjects. MiR-361-3p overexpression suppressed serotonin-induced cell proliferation of hPASMCs by decreasing SERT protein level (Fig. 5c). Our findings provide new insights into the regulatory mechanisms underlying PAH progression.

## Supplementary information

**Additional file 1.** The miR-361-3p level determined using quantitative RT-PCR. hPASMCs were divided into four groups: blank (no treatment), serotonin (treated with 250 μmol/l serotonin for 48 h), serotonin + miR-NC (after transfection with miR-NC for 24 h, treated with 250 μmol/l serotonin for 48 h), and serotonin + miR-361-3p group (after transfection with miR-361-3p mimic for 24 h, treated with 250 μmol/l serotonin for 48 h).

## Data Availability

All data from this study are available in this published article.

## Abbreviations

PAH: Pulmonary arterial hypertension
hPASMCs: Human pulmonary artery smooth muscle cells
5-HT: 5-Hydroxytryptamine
EdU: 5-Ethynyl-2′-deoxyuridine
SERT: Serotonin transporter
miRNAs: MicroRNAs
miR-NC: Negative control miRNA
PVDF: Polyvinylidene difluoride
UTR: Untranslated region
