# Supplementary figures and images for: Upregulation of miR-361-3p suppresses serotonin-induced proliferation in human pulmonary artery smooth muscle cells by targeting SERT
Source: Cell Mol Biol Lett. 2020 Oct 7;25:45. doi: 10.1186/s11658-020-00237-6 (PMC7542879; doi:10.1186/s11658-020-00237-6)

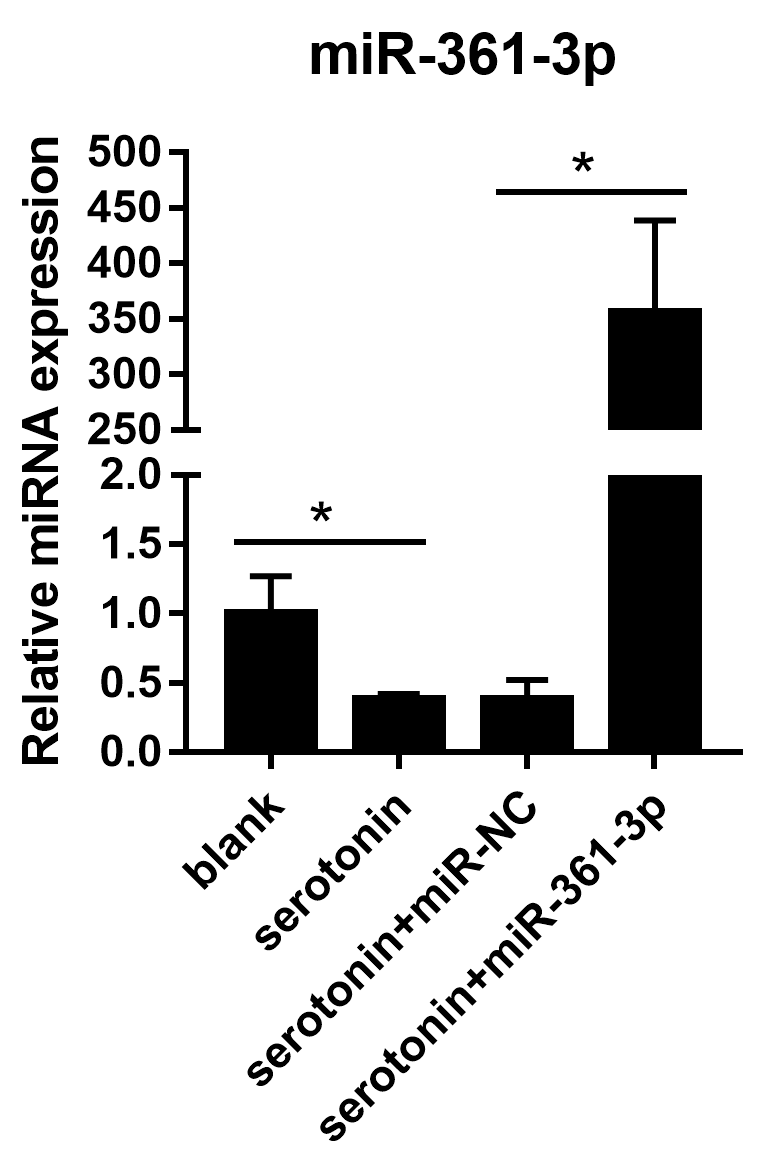

Supplement: Supplementary file 1 — Additional file 1. The miR-361-3p level determined using quantitative RT-PCR. hPASMCs were divided into four groups: blank (no treatment), serotonin (treated with 250 μmol/l serotonin for 48 h), serotonin + miR-NC (after transfection with miR-NC for 24 h, treated with 250 μmol/l serotonin for 48 h), and serotonin + miR-361-3p group (after transfection with miR-361-3p mimic for 24 h, treated with 250 μmol/l serotonin for 48 h). [file 11658_2020_237_MOESM1_ESM.tif]
